# Renal hilar pheochromocytoma: a case report

**DOI:** 10.4076/1757-1626-2-6416

**Published:** 2009-06-29

**Authors:** Youness Ahallal, Mohammed Fadl Tazi, Hind Elfatemi, Kaoutar Znati, Elmehdi Tazi, Afaf Amarti, Mohammed Jamal El Fassi, Hassan Farih M H Moulay

**Affiliations:** 1 Department of Urology, Hassan II Teaching HospitalFezMorocco; 2 Department of Pathology, Hassan II Teaching HospitalFezMorocco; 3 National Institute of Oncology, IbnSina Teaching HospitalRabatMorocco

## Abstract

Paraganglioma is a rare tumor arising from undifferentiated cells of the primitive neural crest. These tumors are most commonly found in the adrenal gland, other localisations are also possible. A 58-year-old woman who presented with history of left lumbar pain, headache, hypertension, palpitation and sweating was found to have a secreting left renal hilar pheochromocytoma. Radical excision of the tumor was therefore undertaken and her hypertension was controlled. From the case report and literature review, the authors suggest a diagnostic and therapeutic strategy for the management of ectopic localization of pheochromocytoma.

## Introduction

Pheochromocytoma is a neuroendocrine tumor that usually develops upon the chromaffin cells of the adrenal medulla. Extraadrenal retroperitoneal paragangliomas arise from paraganglia, which are collections of specialized neural crest cells symmetrically distributed along the aorta in close association with the sympathetic chain. The usual signs are lumbar pain, hypertension associated with generalized symptoms due to raised catecholamines such as headache, blurred vision, heart palpitation and flushing. However, 40% of the retroperitoneal pheochromocytomas don,’t feature any hormonal activity [1].

Following an observation of a renal hilar pheochromocytoma together with a review of the literature, this paper aims at reminding the clinical, therapeutic and histological features of this rare tumor.

## Case presentation

**Figure 1.**
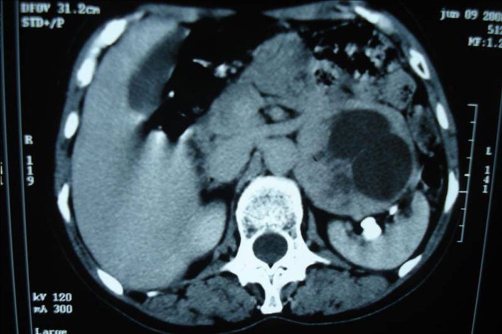
CT scan at level of left renal vein shows a heterogeneous soft-tissue mass.

The patient, a 58-year-old Moroccan housewife, suffered from a 5 year history of left lumbar pain, headache, hypertension, palpitation and sweating. During the physical examination, her blood pressure showed a 200/150 mmHg; she also had a mass in the left subcostal region. The rest of the physical examination was normal. Ultrasonography performed on the patient revealed a 6 cm size heterogeneous mass located in the region of the left renal hilum and an additional computed tomography of the abdomen demonstrated a dumbbell-shaped tumor, measuring 7.0 × 6.5 × 7.5 cm, in the left renal hilum and surrounding the left renal vein with a heterogeneously increasing density after injection of iodine-containing contrast solution. Both adrenal glands were normal with a negative extension summary (Figure 1). Levels of catecholamine metabolites (metanephrine and normetanephrine) in 24-hour urine samples were elevated. The usual paraclinic examinations didn’t display any anomaly. The patient underwent preoperative blockade with phenoxybenzamine, atenolol and metyrosine for 2 weeks.

**Figure 2.**
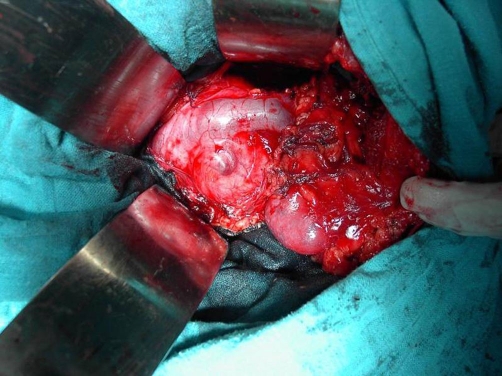
Intraoperative view showing a mass located behind the renal vein.

**Figure 3.**
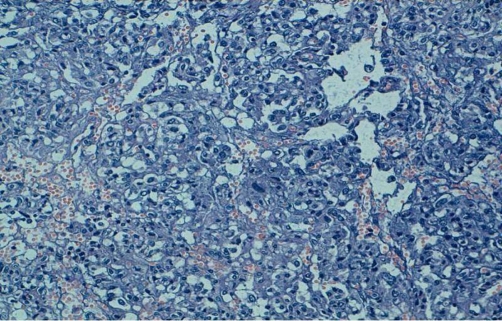
Tumoral proliferation with lobular architecture associated to endocrine vascularisation. (HES × 20)

**Figure 4.**
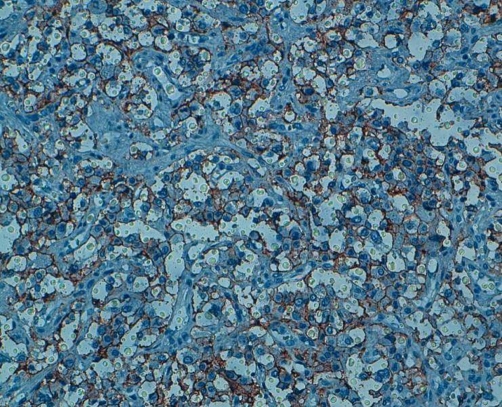
Tumoral cells showing a positive immunostaining for anti-CD56 antibody.

Transperitoneal laparototomy has been therefore performed on the patient; a circumferential dissection of the hilar lesion demonstrated a smooth tumor surrounding the left renal vein (Figure 2). Radical excision of the tumor was undertaken. Pathological examination demonstrated a pheochromocytoma and the surgical margins of the excised specimen were free of tumor (Figures 3 and 4). The patient’s postoperative recovery was smooth and without complications. At the 3-month follow-up, her hypertension was controlled and the urinary catecholamine levels were normal.

## Discussion

Pheochromocytoma develops from the germinal cells coming from the neural crest, and is usually an adrenal tumor. Paragangliomas of the retroperitoneum arise from specialized neural crest cells distributed along the aorta in association with the sympathetic chain. Men are affected more frequently than women, and most patients are between the ages of 30 and 40 years [2,3].

Paragangliomas can be detected early if clinical findings caused by excess secretion of catecholamines are present. The literature states that extraadrenal retroperitoneal paragangliomas are functional for 60% of patients at most [1,3,4]. Characteristic symptoms are sharp headaches, hypertension, palpitation and sweating. For those patients with nonfunctional extraadrenal retroperitoneal paragangliomas, diagnosis usually depends on nonspecific factors related to the growth of a retroperitoneal mass [1,5,6]. The lumbar pain is one of the suggesting signals. The clinical examination can put the light on a lumbar mass. The above mentioned clinical table imposes a measurement of catecholamines and catecholamine metabolites (metanephrine and normetanephrine) in plasma and 24-hour urine samples.

60% of the retroperitoneal pheochromocytomas display signs of hormonal activity; however the hormonal measurement can sometimes be normal [1,3,4].

With regards to the patient mentioned above, the urinary catecholamines measurement was elevated and the radiological diagnosis performed via ultrasonography and computed tomography (CT) maintain the tissue nature of the tumor, which increase its density after iodine contrast solution injection. These two examinations give the opportunity to also seek for concomitant locations, as well as potential ganglionic or visceral metastasis. Computerized tomography is considered as the investigation of choice for demonstrating retroperitoneal structures; it might define the location, extent and nature of these tumors, and may also demonstrate infiltration into surrounding tissues, which provides essential information as to whether the tumor is respectable [7]. Correlation of symptoms and laboratory values of catecholamines is the most efficient way of identifying a soft-tissue retroperitoneal mass, in case it was detected by CT as an extraadrenal retroperitoneal paraganglioma. With nonfunctional paragangliomas, the CT features of extraadrenal paragangliomas overlap those of other retroperitoneal neoplasms, and the diagnosis is more difficult [8]. Percutaneous aspiration cytology may be performed under computerized tomography guidance to obtain a preoperative tissue diagnosis.

The Magnetic Resonance Imaging (MRI) is a very reliable examination that can also detect other localizations despite their small size. Thanks to an outstanding characterization of the tissues, especially at the level of the retroperitoneum and the vascular axes, the added value of the MRI is greater than the one of the CT in the evaluation of the paragangliomas [9]. In order to enhance the diagnosis and the localization of the paragangliomas, a new imaging method has been recently brought forth, using the iodine-131-MIBG scanning [10]. This examination has a sensitivity of 78% to the adrenal pheochromocytoma, and varies in the range from 67% to 89% when it comes to extra-adrenal localizations. It also takes all sense when several localizations are investigated and for the purpose of bone metastasis detection. Its use is very limited though, because of its high cost. The preponderant location of extraadrenal retroperitoneal paraganglioma was infrarenal, as described in previous reports [1,3]. This area, known as the organ of Zuckerkandl, encompasses all chromaffin-cell-bearing tissue along the lower abdominal aorta, its bifurcation, and the iliac vessels [4]. 2 cases of that location of the tumor as reported in our case in the left renal hilum has been already described in the literature.

The treatment of the retroperitoneal pheochromocytoma is solely surgical and the patient preparation is a necessary step and covers a preoperative treatment with α- and β-blocking agents. The treatment of choice is radical excision of the tumor: Krugger-Baggesen et al. reported that out of 15 patients who had undergone a radical operation, none had died of the disease; one patient had nonetheless recurrent disease 7 years later [11]. Radiotherapy and chemotherapy have limited effectiveness.

A long term follow up with regular biological and clinical examinations is necessary, in order to detect any recurrence or metastasis. From a histological perspective, the usual criteria of malignancy have no ground, but the appearance of a metastasis is a sign of malignancy.

Paragangliomas are slow-growing tumors which metastasize late. Long-term survival is expected even with extensive local invasion. Radical excision should therefore be attempted, and if achieved, a good prognosis can be given.

## Conclusion

The renal hilar pheochromocytoma is a rare tumor. Its diagnosis is suspected in the presence of paroxysmal hyper blood pressure paired with a lumbar mass. The treatment of choice of those uncommon tumors is the radical excision that benefits from a good prognostic.

## Abbreviations

CT: Computer tomography
MRI: Magnetic resonance imaging
